# Protective role of Cav-1 in pneumolysin-induced endothelial barrier dysfunction

**DOI:** 10.3389/fimmu.2022.945656

**Published:** 2022-07-27

**Authors:** Robert K. Batori, Feng Chen, Zsuzsanna Bordan, Stephen Haigh, Yunchao Su, Alexander D. Verin, Scott A. Barman, David W. Stepp, Trinad Chakraborty, Rudolf Lucas, David J. R. Fulton

**Affiliations:** ^1^ Vascular Biology Center, Medical College of Georgia, Augusta University, Augusta, GA, United States; ^2^ Department of Forensic Medicine, Nanjing Medical University, Nanjing, China; ^3^ Department of Pharmacology and Toxicology, Medical College of Georgia, Augusta University, Augusta, GA, United States; ^4^ Division of Critical Care and Pulmonary Medicine, Medical College of Georgia, Augusta University, Augusta, GA, United States; ^5^ Department of Phyiology, Medical College of Georgia, Augusta University, Augusta, GA, United States; ^6^ Institute of Human Microbiology, Justus-Liebig University, Giessen, Germany

**Keywords:** caveolin-1, pneumolysin, calcium-influx, barrier-function, endocytosis

## Abstract

Pneumolysin (PLY) is a bacterial pore forming toxin and primary virulence factor of *Streptococcus pneumonia*, a major cause of pneumonia. PLY binds cholesterol-rich domains of the endothelial cell (EC) plasma membrane resulting in pore assembly and increased intracellular (IC) Ca^2+^ levels that compromise endothelial barrier integrity. Caveolae are specialized plasmalemma microdomains of ECs enriched in cholesterol. We hypothesized that the abundance of cholesterol-rich domains in EC plasma membranes confers cellular susceptibility to PLY. Contrary to this hypothesis, we found increased PLY-induced IC Ca^2+^ following membrane cholesterol depletion. Caveolin-1 (Cav-1) is an essential structural protein of caveolae and its regulation by cholesterol levels suggested a possible role in EC barrier function. Indeed, Cav-1 and its scaffolding domain peptide protected the endothelial barrier from PLY-induced disruption. In loss of function experiments, Cav-1 was knocked-out using CRISPR-Cas9 or silenced in human lung microvascular ECs. Loss of Cav-1 significantly enhanced the ability of PLY to disrupt endothelial barrier integrity. Rescue experiments with re-expression of Cav-1 or its scaffolding domain peptide protected the EC barrier against PLY-induced barrier disruption. Dynamin-2 (DNM2) is known to regulate caveolar membrane endocytosis. Inhibition of endocytosis, with dynamin inhibitors or siDNM2 amplified PLY induced EC barrier dysfunction. These results suggest that Cav-1 protects the endothelial barrier against PLY by promoting endocytosis of damaged membrane, thus reducing calcium entry and PLY-dependent signaling.

## 1 Introduction

*Streptococcus pneumoniae*, or pneumococcus, is a Gram-positive bacterium that is the primary etiological agent of community-acquired pneumonia and its associated life-threatening complication, acute respiratory distress syndrome (ARDS) (1, 2). ARDS is defined as an acute inflammatory lung injury associated with increased pulmonary vascular permeability and severe hypoxemia (3). Even with the use of potent and effective antibiotics, mortality from bacterial pneumonia-induced ARDS remains unacceptably high and a greater understanding of the mechanisms underlying pneumococcus-induced lung injury is imperative to foster the development of novel therapeutic approaches.

The primary virulence factor of *Streptococcus pneumoniae* is the cholesterol-dependent cytolysin, pneumolysin (PLY). PLY is a cytoplasmic monomer of 53-kDa that lacks a signal sequence (4). PLY is produced by all clinical isolates of *S. pneumoniae* and is released by break down of the peptidoglycan layer (5, 6) following activation of the cell wall-bound amidase LytA (7, 8) or due to bactericidal antibiotics (1, 9). Alternatively, the peptidoglycan layer can undergo acute structural changes (6) or release extracellular vesicles containing PLY (3).

The cytotoxic effects of PLY are attributed to its potent pore-forming ability in cholesterol rich microdomains of cell membranes. The binding of PLY monomers induces cholesterol-dependent structural changes through oligomerization of monomers resulting in the assembly of transmembrane pores (10, 11). The size of PLY pores is variable (up to 240 Å) and dependent on the amount of PLY present, which can influence cell fate. High concentrations of PLY result in target cell-lysis (12). Whereas sub-lytic concentrations can have a wide range of effects on cell signaling and function including elevation of intracellular Ca^2+^, activation of PKCα (11), myosin light chain kinase (13), ROS production (14), altered RhoA/Rac signaling (15), induction of endothelial contraction and even apoptosis (16, 17). Binding of PLY to endothelial monolayers can disrupt the endothelial barrier and contribute to ARDS.

The distribution of cholesterol in cellular membranes is non-uniform and tightly regulated. Membrane cholesterol is concentrated in specialized microdomains of the plasma membrane called lipid rafts (18). Caveolae are a type of lipid raft that instead of being flat, are small 50-100 nm-wide plasma membrane invaginations. Functionally, caveolae have important cellular roles, including cholesterol homeostasis (19), endocytosis, transcytosis (20, 21) and signal-transduction (22). Caveolins (Cav) are the main structural components of caveolae and expression of Cav family members is tissue-specific, with Cav-1 and -2 expressed in almost every tissue, while Cav-3 is present in skeletal muscle and heart (23). Endothelial cells (ECs) have the highest Cav-1 expression, however, it is also present in adipocytes, smooth muscle cells and fibroblasts (23). Multiple studies have shown that Cav-1 is essential to the formation and stability of caveolae and in cells lacking Cav-1, caveolae fail to form (24, 25). Cav-1 has three major structural domains, a C-terminal membrane attachment domain, a transmembrane domain, and a scaffolding domain (26). Cav-1 has been shown to interact with a wide range of signaling molecules *via* its scaffolding domain, including G-proteins, Src-family tyrosine kinases, integrins, growth factor receptors, endothelial nitric oxide synthase (eNOS) (27), and also with molecules involved in the regulation of IC calcium homeostasis (22).

Recent studies have shown that nucleated cells can actively protect themselves from PLY induced cellular damage *via* elimination of the affected membrane area in microvesicles (28) and that the vast majority of the microvesicle-associated PLY pores are inactive (29). In the present study, we investigated a new mechanism of cellular protection against PLY using an integrated approach of CRISPR-Cas-9 mediated gene editing, gene silencing and overexpression. Collectively, our results indicate that Cav-1 has an important protective role against PLY induced disruption of intracellular Ca^2+^ homeostasis and endothelial barrier dysfunction by promoting the endocytosis of damaged membrane domains.

## 2 Materials and methods

### 2.1 Pneumolysin

LPS-free Pneumolysin (PLY) was purified from a recombinant *Listeria innocua* 6a strain expressing PLY in the laboratory of TC (Justus Liebig University Giessen, Germany). The batch of PLY used in these studies had a specific activity of 1.25 10^7^ hemolytic units/mg.

### 2.2 Cell culture and transfection

COS-7 cells were grown in Dulbecco’s modified Eagle’s medium (DMEM) containing 100 U/mL penicillin, 100 mg/mL streptomycin, and 10% fetal bovine serum (FBS). Lenti-X™ HEK 293T cell line was purchased from Takara Bio USA Inc. (Mountain View, CA) and cultured in DMEM containing 100 U/mL penicillin, 100 mg/mL streptomycin, and and 10% heat inactivated fetal bovine serum (FBS). Primary Human Lung Microvascular Endothelial Cells (HLMVECs) were purchased from Lonza and grown in Endothelial Growth Medium-2-Microvessel (EGM-2MV) containing the requisite growth factors and 5% (FBS) (Lonza, Allendale, NJ). HLMVECs were utilized for experiments between passages 4–12. Immortalized HULEC-5α cells, which overexpress the Simian virus 40A large T antigen, were purchased from the American Type Culture Collection (ATCC, Manassas, VA) and grown in MCDB131 media supplemented with 10% FBS, 10 mM L-glutamine, 1 μg/mL hydrocortisone, and 10 ng/mL Epidermal Growth Factor (EGF). All cells were cultured at 37°C, 5% CO_2_ in a humidified atmosphere. For transfection, COS-7 and HEK293 cells were transfected using Lipofectamine 3000 (Invitrogen, Grand Island, NY) according to the manufacturer’s instructions. Adenoviral constructs were generated in house, amplified in HEK293 cells, purified by CsCl gradient ultracentrifugation and dialyzed and stored at -80 °C as we have shown previously (30).

### 2.3 Gene silencing

For gene silencing experiments HLMVEC or HULEC5α cells were seeded on 6 well plates and transfected with non-specific (scrambled), or caveolin-1 specific siRNA at 20 nM final concentration using the Lipofectamine™ RNAiMAX transfection reagent, according to the manufacturer’s instructions. After 3 hours of incubation, transfection media was replaced with complete media supplemented with 10% FBS final concentration, followed by 48 h further incubation, and the cells were incubated for 72 hours at 37°C.

### 2.4 Cholesterol depletion and supplementation

To deplete or load cholesterol into the plasma membrane of COS-7 cells, cells were seeded at a density of 2.5×10^5^ cells/3.8 cm^2^. To extract membrane cholesterol, cells were incubated in serum-free medium containing 10 mM methyl-β-cyclodextrin (MβCD). To load cholesterol into cells, MβCD (10 mM) was dissolved in warm DMEM and cholesterol was dissolved in ethanol and then combined to make a final concentration of 10 mM MβCD + 2 mM cholesterol (5:1) solution. Cholesterol was subsequently loaded into membranes by incubation for 0.5 - 1 hour at 37 °C.

### 2.5 Measurements of intracellular Ca^2+^

Intracellular calcium levels were measured either with using calcium indicator Fluo-4 AM (Molecular Probes) according to the manufacturer’s instructions or with the calcium-activated photoprotein, aequorin, as described previously (15). In brief, for aequorin-mediated fluorescence measurements COS-7, HULEC5α or Cav-1 KO HULEC5α cells were transduced with adenoviruses encoding aequorin, RFP or Cav-1 at 15 MOI, and 24 h later the aequorin was reconstituted by treating cells with 5 μM coelenterazine (Sigma, St. Louis, MO) for 1 hour in serum-free and phenol free DMEM containing 0.1 mM EDTA. Then, the cells were exposed to different concentrations of PLY in Hank’s Balanced Soult Solution (HBSS) for 10 minutes, and luminescence was recorded using a PolarSTAR luminometer (BMG Labtech, Cary, NC). After the baseline luminescence values were recorded, calcium chloride was injected at a 1.8 mM final concentration to enable the measurement of calcium induced changes in luminescence which reflect calcium influx.

### 2.6 Western blotting analysis

Cells were washed once with ice cold PBS on ice, then lysed in 2x Laemmli Sample buffer, scraped and sonicated. All of the samples were boiled for 6 minutes at 100°C and analyzed by Western blotting. Protein samples were separated by 10% SDS-PAGE and transferred to 0.20 µM pore size nitrocellulose membrane with Trans-Blot^®^ Turbo™ Transfer System, 1.3 A, 25V for 10 minutes. The membranes were then blocked with 5% (w/v) nonfat dry-milk powder solution in TBS-Tween 20 (TBST) and incubated with specific primary antibodies overnight at 4°C. The primary antibodies used in this study were as follows: anti-Hsp90 (Cell Signaling Technology, Cat# 4874), anti-caveolin-1 (Cell Signaling Technology Cat# 3238), β-tubulin (BD Biosciences, Cat# 556321), anti-dynamin II (BD Biosciences, Cat# 610263). After incubation with the primary antibodies, the membranes were washed three times for 10 minutes with TBST and incubated with horseradish-peroxidase (HRP) conjugated anti-mouse (Cell Signaling Technology, Cat# 7076) or anti-rabbit (Cell Signaling Technology, Cat# 7074) secondary antibodies. Immunoreactive proteins were visualized by enhanced chemiluminescence (ECL) using autoradiography films. ImageJ software (Research Services Branch, National Institute of Health (Bethesda, MD) was used for densitometric analyses.

### 2.7 Endothelial permeability measurements *via* electric cell substrate impedance sensing

As an index of cellular permeability and barrier function, the transendothelial electrical resistance (TER) of HLMVECs or HULEC5α was measured using electric cell-substrate impedance sensing (ECIS) in a ECIS System Model 1600R (Applied Biophysics, Carlsbad, California). In brief, equal numbers of cells (3 x 10^4^/well) were seeded on 8W10E ECIS arrays, and measurements were obtained when resistance values of the cells reached ≥1000 Ω of steady-state resistance at a frequency of 4000 Hz. In some experiments, 12 hours after plating EC into ECIS arrays, the cells were treated either with Cav-1 peptide (Enzo Life Sciences, Cat# ALX-153-064-M001), Cav-1 adenovirus (10 MOI), RFP adenovirus (10MOI) or vehicle for an additional 24 hours. Cells were then challenged with PLY or vehicle in serum free media. Data were normalized to the initial resistance values and plotted as normalized resistance.

### 2.8 Generation of CRISPR-Cas9 mediated Cav-1 knockout microvascular endothelial cells by lentivirus mediated gene delivery

#### 2.8.1 Lentivirus generation

Lentivirus-containing supernatants were generated using Lipofectamine 3000 mediated transfection of the Lenti-X™ 293T packaging cells line and co-transfection of the vector plasmid pTLCV2 (Addgene #87360) encoding a TET-on Cas9-2A-eGFP and one of four guide RNAs (see **Table 1**), the 2^nd^ generation packaging plasmid psPAX2 and the envelope expressing plasmid pMD2.G (Addgene cat#87360, #12260 and #12259) in 2:6:4 ratio, respectively. In brief, the day before transfection Lenti-X™ 293T cells were plated in 150 mm cell culture dishes and grown overnight to reached ~80% confluence. Next, cells were treated with 25 μM chloroquine diphosphate dissolved in complete DMED culture media for 5 hours to decrease endogenous nuclease activity. After chloroquine treatment the cells were transfected with a total of 12 μg plasmid (endotoxin free)-lipofectamine complex per plate in Opti-MEM for 3 hours according to the manufacturer’s instructions. Next, the transfection media was replaced with DMEM supplemented with 10% heat inactivated FBS, 2 mM caffeine, 100 U/mL penicillin, 100 mg/mL streptomycin and the cells were incubated for an additional 48 hours. The media was then collected and resulting lentiviruses further concentrated.

**Table 1:** Guide RNA sequences.

**Guide 1 (Exon 1)**	
Forward: CACCGCCACGGGCCAGCATGTCTGG
Reverse: AAACCCAGACATGCTGGCCCGTGGC
**Guide 2 (Exon 2)**	
Forward: CACCGAGTGTACGACGCGCACACCA
Reverse: AAACTGGTGTGCGCGTCGTACACTC
**Guide 3 (Exon 3)**	
Forward: CACCGTCACTGTGACGAAATACTGG
Reverse: AAACCCAGTATTTCGTCACAGTGAC
**Guide 4 (Exon 2)**	
Forward: CACCGTAAACACCTCAACGATGACG
Reverse: AAACCGTCATCGTTGAGGTGTTTAC

#### 2.8.2 Lentivirus concentration

Concentration of lentivirus has been previously described (31) and was performed with minor modifications. Lentivirus containing supernatants were pre-clarified by centrifugation at 900g for 10 minutes at 4 °C and then supernatants passed through a 0.45 μm filter. Pre-clarified supernatants were then overlaid on a sucrose buffer (50 mM TRIS-HCl pH 7.4, 100 mM NaCl, 0.5 mM EDTA, 10% sucrose) in 4:1 ratio in a 50 mL polypropylene tube and centrifuged at 4910g for 4 hours at 4 °C. Following centrifugation, the supernatant was carefully aspirated and the 50 mL tubes were inverted on a paper towel for 3 minutes. Next, 300 μl PBS was added to the tubes, and the tubes were kept at 4 °C overnight. 24h later, resuspended lentiviral particles were aliquoted, snap-frozen and stored at -80 °C.

#### 2.8.3 Cav-1 KO HULEC-5a cell line generation

In order to generate Cav-1 knock-out immortalized lung microvascular EC, HULEC-5a cells were first plated in 12 well plates. At ~ 40% confluence, the cells were transduced either with the Guide-4 coding lentivirus alone targeting exon-2, or with a combination of all four lentiviruses targeting exon-1, -2 and -3 of the Cav-1 gene and the cells were incubated for 6 hours in the presence of 10 μg/mL polybrene (Sigma-Aldrich Corp., St. Louis, MO) or TransDux™ (System Biosciences, Palo Alto, CA). The supernatant was then removed and the cells allowed to recover for 24h in complete MCDB-131 media, and then treated with 1 μg/mL doxycycline either for 1-3 days in time course experiments, or at least for four days in all other experiments and GFP expression monitored by microscopy. After doxycycline treatment, antibiotic selection (puromycin, 1 μg/ml) was applied for 48h, and surviving cells were used for further experiments when cultured in the presence of puromycin (0.25ug/ml).

### 2.9 Statistical analysis

Data are expressed as means ± SEM and statistical analyses were performed using GraphPad Software (San Diego, CA) with a student t-test or two-tailed ANOVA with a *post hoc* test where appropriate. Statistical significances were considered as p < 0.05.

## 3 Results

### 3.1 Caveolin-1 protects against PLY-induced calcium entry and endothelial barrier dysfunction

**Figure 1:**
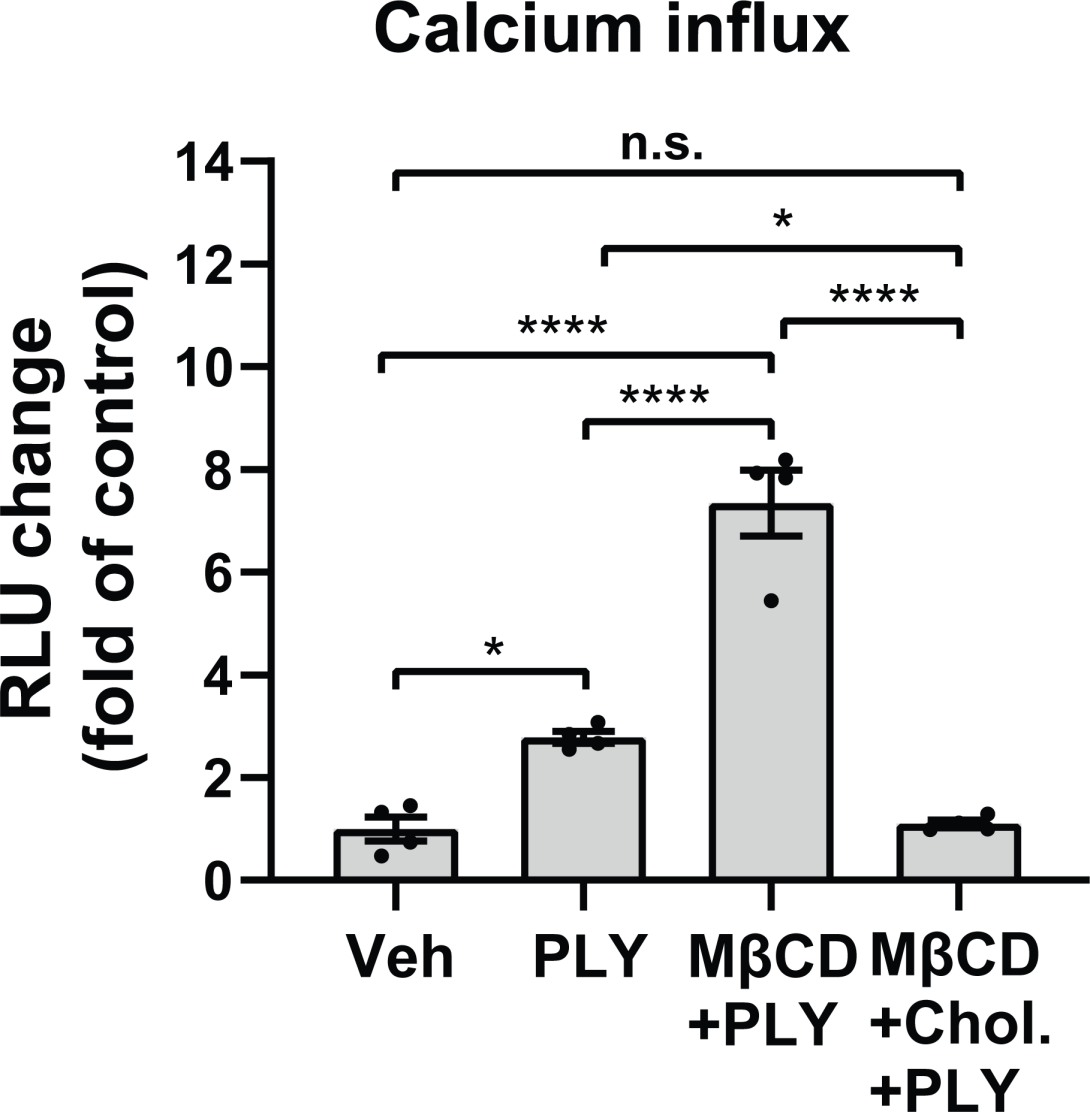
Membrane cholesterol paradoxically inhibits PLY-induced calcium-entry. Membrane cholesterol was either depleted (MβCD) or loaded (MβCD+Chol.) into the plasma membrane of COS7 cells for 30 minutes, followed by a 10 minutes treatment with vehicle (Veh) or PLY (60 ng/mL) and changes of intracellular calcium levels were measured by aequorin-mediated luminescence as described in materials and methods. Data are represented as mean ± SE, n.s., not significant, *P<0.05, ****P<0.0001 (n=4). One-way ANOVA (with Tukey’s *post-hoc* test).

**Figure 2:**
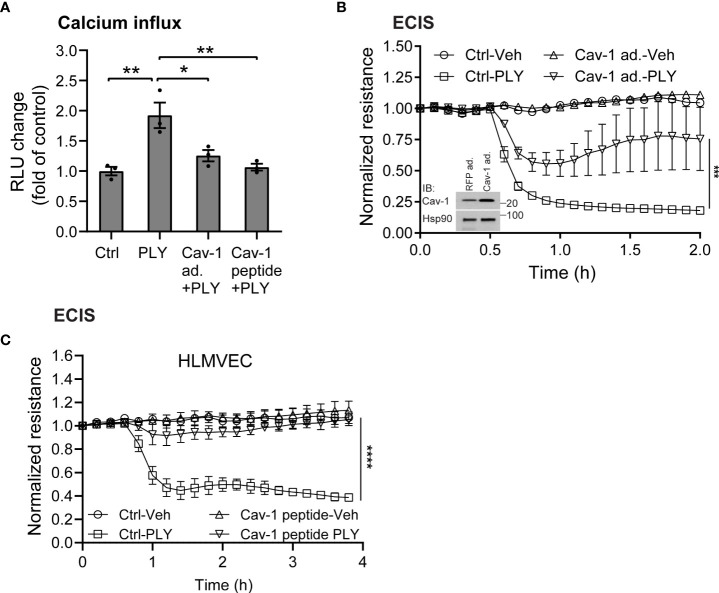
Caveolin-1 overexpression protects against Pneumolysin (PLY) induced calcium entry and endothelial barrier dysfunction. **(A)** Caveolin-1 overexpression or Cav-1 peptide blocks PLY induced Ca^2+^ entry in Cos7 cells. Data are represented as mean ± SE (n=3-4), * P<0.05, **P<0.01 vs. vehicle control. **(B)** HLMVEC cells were transduced with 10-10 MOI RFP (RFP ad.) or caveolin-1 adenovirus (Cav-1 ad.) (*Inset*: western blot analysis of Caveolin-1 overexpression. Hsp90 was used as loading control), or **(C)** treated with 10 μM caveolin-1 scaffolding domain peptide for 24 hours, and TER of vehicle or PLY (60ng/mL) treated HLMVECs was measured (n=3). ***P<0.001, ****P<0.0001. One-way ANOVA (with Tukey’s *post-hoc* test).

Several studies have shown that the pneumococcal toxin, PLY, preferentially binds to cholesterol-rich membrane domains to induce pore formation (12, 32, 33), leading to an increase in calcium influx (11). Consistent with these findings, herein, we show that PLY treatment of COS-7 cells resulted in a significant increase in IC Ca^2+^ (**Figure 1**). Since PLY is a cholesterol-dependent toxin, we next tested the hypothesis that depletion of cholesterol with MβCD would reduce Ca^2+^ entry. Surprisingly, MβCD treatment did not reduce PLY-induced Ca^2+^ influx. In contrast, we observed a significant increase in intracellular Ca^2+^ levels (mean 7.34 **±** 0.55**)**. When cholesterol was loaded into the cell membranes using the combination of MβCD and cholesterol, PLY-induced intracellular Ca^2+^ influx was reduced (mean = 1.09 **±** 0.06) (**Figure 1**). The ability of MβCD to deplete plasma membrane cholesterol has an **“**anti-caveolin-1**”** activity, since it is known to disrupt the structure of caveolae (34). Because Cav-1 is critical to caveolae biogenesis (35) which can impact signaling and calcium-entry, we next tested the effect of Cav-1 overexpression on Ca^2+^ influx (**Figure 2**). We found that overexpression of Cav-1 and pretreatment of cells with the Cav-1 scaffolding domain peptide significantly decreased PLY-induced Ca^2+^ entry (**Figure 2A**). Next, we investigated the effect of Cav-1 overexpression on microvascular endothelial permeability using ECIS measurements. We found that adenovirus-mediated overexpression of Cav-1 (**Figure 2B**) significantly decreased the barrier-disruptive effects of PLY. Similar effects were observed when the cells were treated with the Cav-1 scaffolding domain peptide (cavtratin) (**Figure 2C**).

### 3.2 Loss of Cav-1 amplifies the effects of PLY on endothelial cells

**Figure 3:**
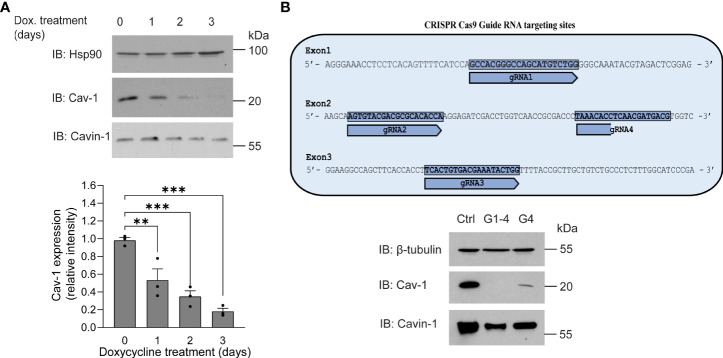
Generation of caveolin-1 knock-out HULEC5α cells by CRISPR-Cas9 lentiviral system mediated gene editing. **(A)** HULEC5α cells were simultaneously transduced by lentiviruses encoding four different guide RNAs (G1-4) targeting exons 1-4 of Cav-1. After 6 hours, cells were treated for four days with 1 µg/mL doxycycline to induce Cas9 and loss of caveolin-1 through genome editing. After administration of doxycycline, Cav-1 and cavin-1 expression was monitored for three days. Representative immunoblots are showing a time dependent decrease of Cav-1 (n=3), where Hsp90 was used as loading control. **(B)** *Upper panel*: schematic representation of gRNA targeting strategy. *Lower* panel: When only one guide RNA was used (G4), Cav-1 expression was reduced but not to the extent of multiple guides. Representative immunoblots showing the expression of Cav-1 and cavin-1 (n=3). β-tubulin was used as loading control. **P<0.005, ***P<0.001. One-way ANOVA (with Tukey’s *post-hoc* test).

The inverse correlation between higher expression levels of Cav-1 or its scaffolding domain and the reduced effects of PLY on calcium influx and endothelial barrier function prompted us to investigate whether the loss of Cav-1 can increase susceptibility to PLY. To reduce Cav-1 expression we generated Cav-1 knockout immortalized lung microvascular endothelial cells (Cav-1 KO HULEC5α) using doxycycline inducible CRISPR-Cas9 mediated gene editing (**Figure 3**). In time course experiments, we have found that three days of doxycycline exposure was sufficient to decrease the level of Cav-1 by more than 82% on average (day 3: mean = 0.1819 ± 0.028) (**Figure 3A**). We have also tested the knockout efficiency when only one or all three exons of Cav-1 were targeted and we have found that targeting multiple exons was more efficient at promoting the acute loss of Cav-1 protein expression (**Figure 3B**). The level of cavin-1, another caveolar protein involved in caveolae biogenesis and structure was partially decreased, which is in accord with the findings of Davalos et al. (36)

**Figure 4:**
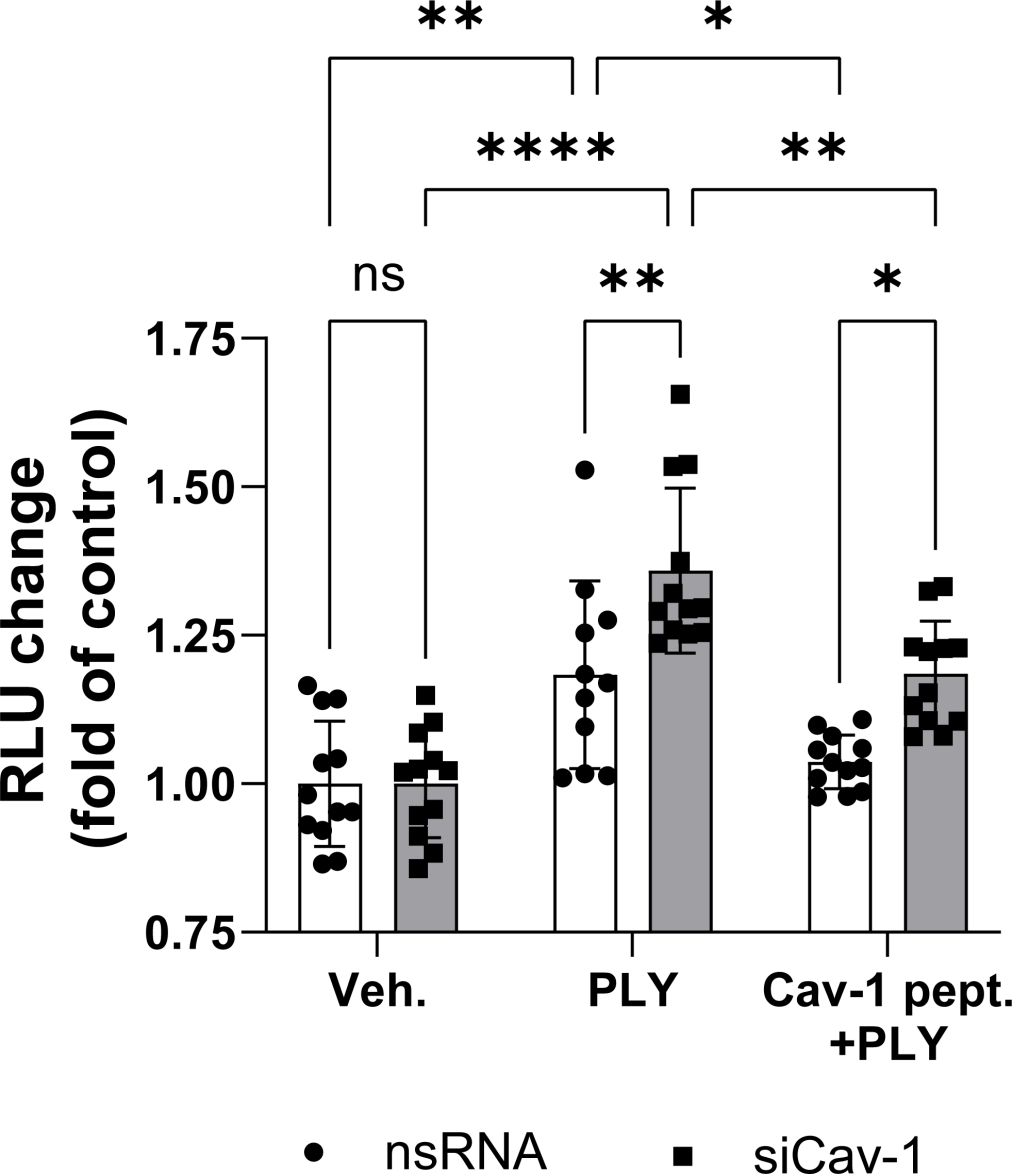
Caveolin-1 protects against pneumolysin induced calcium entry in HULEC5α. Control (vehicle treated) or caveolin-1 silenced HULEC5α cells were treated with PLY (60 ng/mL) for 10 minutes and calcium influx was detected by Fluo-4-mediated fluorescent intensity (n=11-12). ns, not significant, *P<0.05, **P<0.01, ****P<0.0001, one-way ANOVA (with Tukey’s *post-hoc* test).

To assess the functional role of Cav-1 on PLY induced endothelial cell dysfunction we measured intracellular calcium concentrations in control and Cav-1 silenced cells. We found that stimulation of endothelial cells with 60 ng/ml PLY significantly increased intracellular Ca^2+^ levels and this increase was significantly higher in cells lacking Cav-1 (nsRNA + PLY vs. siCav-1 + PLY mean: 1.183 ± 0.045 vs. 1.359 ± 0.038). On the other hand, treating the Cav-1 silenced cells with cavtratin significantly decreased PLY induced calcium influx (siCav-1 + Cav-1 peptide + PLY mean: 1.185 ± 0.025) (**Figure 4**).

**Figure 5:**
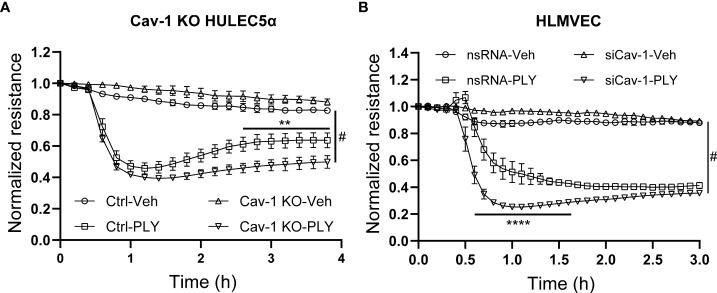
Knock-out or silencing of Cav-1 facilitates PLY induced barrier dysfunction in human lung microvascular endothelial cells. **(A)** Control and Cav-1 knockout HULEC5α cells were treated with PLY and barrier function assessed by changes in TER. (n=3). **P<0.01 control-PLY vs Cav-1 KO-PLY, ^#^P<0.0001 when compared to vehicle control. **(B)** HLMVECs were treated with non-targeting (nsRNA) or caveolin-1 specific silencing RNA (siCav-1) for 72 hours and TER was measured upon challenge with PLY (60 ng/mL), (n=3-4). ****P<0.0001 control-PLY vs siCav-1-PLY, ^#^P<0.0001 when compared to vehicle control. Two-way ANOVA (with Tukey’s multiple comparisons test).

To further asses the functional significance of the loss of Cav-1 we next measured endothelial permeability in ECIS arrays and we have found that the absence of Cav-1 further amplified the PLY induced endothelial barrier disruptive effects in Cav-1 KO HULEC5α cells (**Figure 5A**) and also in Cav-1-silenced HLMVECs (**Figure 5B**).

### 3.3 Re-expression of Cav-1 in Cav-1 knockout cells rescues barrier function

**Figure 6:**
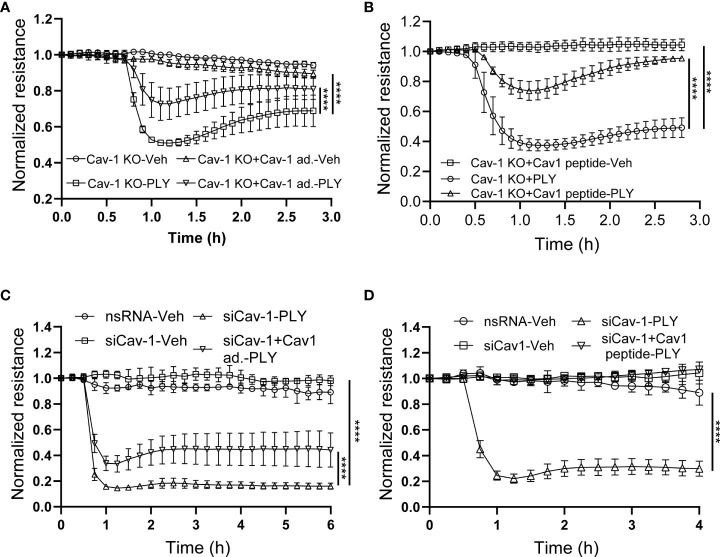
Reconstitution of Cav-1 or its scaffolding domain signaling attenuates PLY-induced endothelial barrier disruption. **(A)** Caveolin-1 overexpression in Cav-1 knock-out HULEC5α cells ameliorates PLY induced endothelial barrier dysfunction. Cav-1 knock-out endothelial cells were transduced with RFP or Cav-1 adenovirus (10 MOI). After 48 hours the cells were treated with either vehicle or PLY (60ng/mL) and barrier function assessed by changes in TER. **(B)** Caveolin-1 scaffolding domain peptide ameliorates PLY induced endothelial barrier dysfunction. Cav-1 knockout HULEC5α were administered the Cav-1 scaffolding domain peptide (10 µM) or vehicle (DMSO). After 24 hours, cells were treated with vehicle (Veh.) or PLY (60 ng/mL) and TER was measured. **(C)** HLMVECs were transfected with non-specific or Cav-1 specific silencing RNA and rescue experiments were performed by transducing Cav-1 silenced cells with RFP or Cav-1 adenovirus. After 24 hours HLMVECs were treated with vehicle (Veh) or PLY (60 ng/mL) and TER was measured. **(D)** HLMVECs were transfected with non-specific or Cav-1 specific silencing RNA and rescue experiments were performed by treating the cells with Cav-1 scaffolding peptide (10 µM) or DMSO for 24 hours, followed by PLY (60 ng/mL). Endothelial barrier function was assessed by changes in TER (n=3). ****P<0.0001, two-way ANOVA (with Tukey’s multiple comparisons test).

An advantage of the CRISPR-Cas9-mediated Cav-1 KO HULEC5α cell line is the ability to perform rescue experiments with Cav-1 re-expression. We found that the re-expression of Cav-1 (**Figure 6A**) and scaffolding domain peptide treatments (**Figure 6B**) were protective against PLY induced barrier disruption. These results were recapitulated in HLMVECs when Cav-1 expression was depleted using siRNA, followed by overexpression of Cav-1 (**Figure 6C**) or treatment of cells with the scaffolding domain peptide (**Figure 6D**).

### 3.4 Cav-1 mediated endocytosis promotes the elimination of PLY pores from the membrane

**Figure 7:**
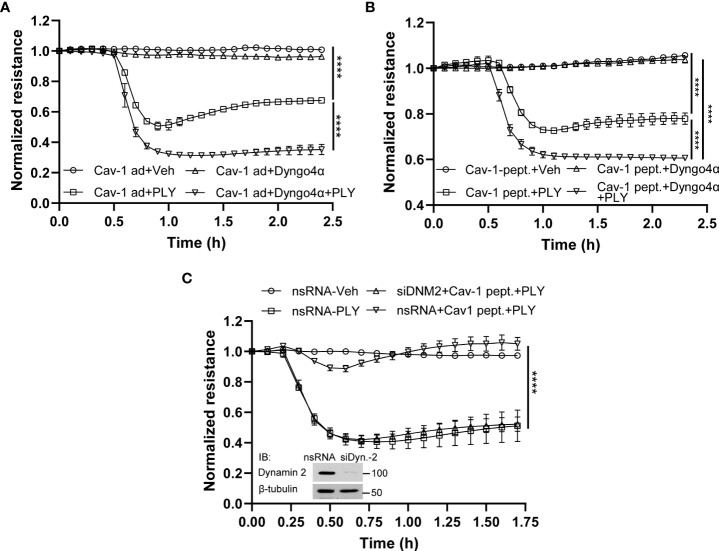
Inhibition of endocytosis attenuates the protective effect of Cav-1 and caveolin-1 scaffolding domain peptide on HULEC5α barrier function against PLY-induced endothelial barrier disruption. **(A)** The inhibitor of dynamin, Dyngo4α, decreased the protective effect of Cav-1 against PLY-induced barrier dysfunction. HULEC5α cells were transduced Cav-1 adenovirus for 24 hours and treated with vehicle or10 µM Dyngo4α for 4 hours prior to PLY (60 ng/mL) administration (n=3). ****P<0.0001, Cav-1 ad + PLY vs. Cav-1 ad.+ Dyngo 4α + PLY. **(B)** The dynamin inhibitor, Dyngo4α, diminishes the protective effect of the Cav-1 scaffolding domain peptide against PLY-induced barrier disruption. HULEC5α cells were transduced with DMSO (Ctrl) or the Cav-1 scaffolding domain peptide (Cav-1 peptide) for 24 hours prior to PLY treatment (n=3). ****P<0.0001. **(C)** Loss of dynamin2 expression reduces the protective effects of the Cav-1 scaffolding domain peptide against PLY-induced barrier disruption. HULEC5α cells were treated with either non-specific or Cav-1 specific siRNA for 24 hours and cells were treated with vehicle (DMSO) or Cav-1 scaffolding domain peptide (Cav-1 peptide) for an additional 24 hours prior PLY (60 ng/mL) treatment. TER changes were monitored over time **(A–C)** (n=3), ****P<0.0001, two-way ANOVA (with Tukey’s multiple comparisons test). (*Inset*: western blot analysis of dynamin-2 silencing, β-tubulin was used as loading control).

To determine the mechanisms by which Cav-1 and its scaffolding domain peptide protect the endothelial barrier from PLY-induced cellular injury, we next investigated the role of caveolin-mediated endocytosis. Studies have shown that Cav-1 mediated endocytosis is cholesterol/raft-dependent and involves dynamin-2 (37). HULEC5α were either transduced with Cav-1 adenovirus or treated with the scaffolding domain peptide (**Figures 7A**, **B**) and PLY was administered in the presence or absence of the highly selective dynamin-2 inhibitor, dyngo4α and barrier function determined by measurement of TER. We found that inhibition of dynamin-2 decreased the barrier protective effects of both Cav-1 and its scaffolding domain peptide.

To more specifically investigate a role for dynamin-2, we used siRNA to silence endogenous dynamin-2 in HULEC5α cells prior to PLY treatment. Consistent with our previous results, the Cav-1 scaffolding domain peptide was highly effective in preventing PLY-induced disruption of the EC barrier in control silenced HULEC5α (**Figure 7C**). However, this protective effect was completely lost in cells lacking dynamin-2. These results indicate that Cav-1 and dynamin-2 have important and interrelated roles in regulating the integrity of the endothelial barrier and its susceptibility to PLY.

## 4 Discussion

The opportunistic pathogen, *S. pneumoniae* is responsible for a wide range of diseases including community-acquired pneumonia. Despite the development of highly effective antibiotics, community-acquired pneumonia continues to be associated with considerable morbidity and mortality worldwide (38). The cholesterol-dependent cytolysin (CDC) PLY is one of the major virulence factors of pneumococcus and it significantly contributes to bacterial penetration, inflammation and direct cell damage *via* its pore-forming activity (38, 39). PLY preferentially binds to cholesterol in the cell membrane which triggers pore assembly and it is generally accepted that cholesterol is a PLY receptor (32). During the process of pore-formation, PLY monomers gather at the surface of cholesterol rich membrane domains and then assemble into a ring-shaped pre-pore complex. Insertion of the PLY pore-forming complex into the lipid bilayer results in the loss of membrane integrity, Ca^2+^ influx and even cell death if the PLY concentration is excessive (12). There are a number of cholesterol-rich membrane domains including lipid rafts and the small, flask shaped membrane invaginations, termed caveolae. Caveolae have important roles in several physiological processes, but also serve as a hub for coordinating outside-in and intracellular signaling pathways (23). The major scaffolding protein of caveolae is the 22 kDa transmembrane protein Cav-1, which is highly expressed in endothelial cells (40). Cav-1 is required for caveolae biogenesis as the organelle is absent in endothelial cells and adipocytes isolated from Cav-1 null mice (41). In addition to its structural role in forming caveolae, Cav-1 has important roles in the regulation of cell proliferation, cell metabolism, vesicular transport, transcytosis and endocytosis (37, 42), potocytosis and inhibition of extracellular receptors such as GCPRs, EGFR and inhibition of several intracellular signaling molecules such as eNOS, G-proteins, Src-kinase, PKCα and various other mechanisms (14, 43–46). Important roles of Cav-1 in the pathogenesis of inflammation (44), fibrosis (47) and acute lung injury (48) are also well established. Given the wide range of cellular functions influenced by Cav-1, the mechanisms by which it influences disease pathways are complex and incompletely understood. The ability of caveolae to internalize bacterial toxins such as cholera and tetanus toxins has long been proposed and it has also been shown that eukaryotic cells can defend against pore forming toxins *via* membrane blebbing and shedding of microvessicles (28, 49). However, the internalization of damaged membrane particles by caveolae-dependent mechanisms has not been reported. In the current study, we show that both Cav-1 and its scaffolding domain peptide can attenuate the Ca^2+^ influx and the subsequent endothelial barrier disruption in response to the pore-forming toxin PLY and this process is mediated by caveolin regulated endocytosis.

Given that PLY binds and inserts into cholesterol-enriched membrane microdomains our initial hypothesis was that depletion of cholesterol from the plasma membrane by MβCD treatment would reduce PLY binding and consequently block Ca^2+^ influx. In contrast, we found that MβCD treatment robustly increased intracellular Ca^2+^ levels in the presence of PLY, and this increase was accompanied by greater disruption of the endothelial barrier as assessed by ECIS measurements. The loss of endothelial barrier integrity in PLY-treated cells is not surprising in the light of recent reports showing that Ca^2+^ influx plays a major role by activating the calcium sensitive PKCα isoform resulting in disassembly of VE-cadherin junctions and increased endothelial permeability (11, 50). Increased levels of intracellular Ca^2+^ also activates myosin light chain kinase (MLCK) which plays an important role in controlling the permeability of microvascular barriers *via* acto-myosin phosphorylation (51). Furthermore, our lab has also shown previously that the related G^+^ bacterial pore-forming toxin, listeriolysin O (LLO), stimulated the PKCα and eNOS-dependent production of superoxide and promoted the dissociation of heat-shock protein 90 (Hsp90) and Cav-1 from eNOS while disrupting endothelial barrier integrity (52). Indeed, our data show that increased expression of Cav-1 or its scaffolding domain peptide is protective upon PLY induced Ca^2+^ influx and barrier disruption, while the lack of Cav-1 in CRISPR-Cas9 edited Cav-1 KO or in Cav-1 silenced cells further amplified these effects. Notably, MβCD treatment not only depletes the cholesterol from the plasma membrane, but also leads to disassembly of caveolae and caveolae-mediated signaling (45, 53), which supports the hypothesis that the barrier disruptive effects of PLY are regulated by caveolae and caveolar proteins.

Our previous studies by Lucas and coworkers (11) have shown that the Ca^2+^ channel antagonist LaCl_3_, dose-dependently inhibits PLY-induced barrier disruption in HLMVEC. Recent studies, however, support an emerging paradigm shift that PLY not only functions as a cholesterol dependent calcium ionophore, but may also have actions on cellular receptors other than cholesterol. It has been shown that PLY stimulates numerous signaling pathways including phospholipase A2 (PLA2) (54) and phospholipase C (PLC) (55, 56). Others have shown that Src-dependent phosphorylation of caveolin-1 Tyr-14 promotes the swelling and endocytosis of caveolae (57) which could reduce inflammatory signaling.

Insertion of large and/or numerous PLY pores into the plasma membrane can elicit a long-lasting elevation of Ca^2+^ that is toxic to the cell (58). However, eukaryotic cells have developed several membrane repair mechanisms to isolate, reseal and eliminate membrane pores (59). It has been reported, that the Ca^2+^ influx resulting from plasma membrane damage triggers membrane repair mechanisms (49, 60), however long-lasting Ca^2+^ elevations are toxic (58). Current models for membrane repair include patch formation, which is the fusion of intracellular vesicles with areas of plasma membrane that are damaged, shedding and endocytosis (49, 60). The most common strategy used to remove an active pore is membrane shedding, while inactive pores are removed *via* endocytosis (49). Both sub-lytic and lytic concentrations of PLY result in plasma membrane pores but how long these pores persist and the mechanisms of pore removal remain incompletely understood (12). Interestingly, caveolin plays an underappreciated role in membrane repair and Cav1-deficient cells have impaired ability to repair pore forming toxin-induced injuries (49).

The internalization of PLY-induced active pores by caveolae-dependent mechanisms has not been investigated and thus the role of endocytosis as a mechanism for PLY pore removal remains controversial. A key regulator of endocytosis is dynamin-2, which is important in the regulation of clathrin- and RhoA-dependent as well as caveolin-mediated endocytosis of which only the latter two are cholesterol dependent (61). In the current study, we provide new evidence that inhibition of endocytosis *via* a highly specific dynamin-2 inhibitor or by siRNA mediated gene silencing of dynamin-2, attenuates the barrier protective effects of Cav-1 and its scaffolding domain peptide. These data suggest that caveolin-mediated endocytosis is important in preserving the barrier integrity against PLY. However, a role of clathrin-mediated endocytosis cannot be excluded either and could be addressed with additional experiments in future studies.

In conclusion, our studies identify a new Cav-1 mediated and dynamin-2 dependent mechanism regulating PLY induced calcium influx and loss of endothelial barrier function. These studies extend our knowledge of the protective mechanisms by which host cells defend cellular integrity against bacterial pore forming toxins and provide new avenues for therapeutic intervention.

## Data availability statement

The original contributions presented in the study are included in the article/supplementary material. Further inquiries can be directed to the corresponding authors.

## Author contributions

Conceptualization and experimental design, RB, FC, ZB, SB and DF. Resources, TC. Data curation, RB, and DF. Writing—original draft preparation, RB and DF. Writing—review and editing, RL, AV, DS, SB and YS. Supervision, DF. All authors have read and agreed to the published version of the manuscript.

## Funding

This research was funded by the National Institute of Health R01HL147159 (DS, DF), R01 HL156646 (SB, DF), R01HL138410 (RL), TPA34910080 (DF) and American Heart Association Postdoctoral Fellowship 20POST35210753 (RB).

## Acknowledgments

We thank Supriya Sridhar and Madison West for their technical assistance, and Sandy Fergusson for her help in administration.

## Conflict of interest

The authors declare that the research was conducted in the absence of any commercial or financial relationships that could be construed as a potential conflict of interest.

## Publisher’s note

All claims expressed in this article are solely those of the authors and do not necessarily represent those of their affiliated organizations, or those of the publisher, the editors and the reviewers. Any product that may be evaluated in this article, or claim that may be made by its manufacturer, is not guaranteed or endorsed by the publisher.
